# Computational design of substrate selective inhibition

**DOI:** 10.1371/journal.pcbi.1007713

**Published:** 2020-03-20

**Authors:** Benny Da’adoosh, Kon Kaito, Keishi Miyashita, Minoru Sakaguchi, Amiram Goldblum

**Affiliations:** 1 Molecular Modeling Laboratory, Institute for Drug Research, The Hebrew University of Jerusalem, Jerusalem, Israel; 2 Laboratory of Cell Biology, Osaka University of Pharmaceutical Sciences, Osaka, Japan; University of Michigan, UNITED STATES

## Abstract

Most enzymes act on more than a single substrate. There is frequently a need to block the production of a single pathogenic outcome of enzymatic activity on a substrate but to avoid blocking others of its catalytic actions. Full blocking might cause severe side effects because some products of that catalysis may be vital. Substrate selectivity is required but not possible to achieve by blocking the catalytic residues of an enzyme. That is the basis of the need for "Substrate Selective Inhibitors" (SSI), and there are several molecules characterized as SSI. However, none have yet been designed or discovered by computational methods. We demonstrate a computational approach to the discovery of Substrate Selective Inhibitors for one enzyme, Prolyl Oligopeptidase (POP) (E.C 3.4.21.26), a serine protease which cleaves small peptides between Pro and other amino acids. Among those are Thyrotropin Releasing Hormone (TRH) and Angiotensin-III (Ang-III), differing in both their binding (K_m_) and in turnover (k_cat_). We used our in-house "Iterative Stochastic Elimination" (ISE) algorithm and the structure-based "Pharmacophore" approach to construct two models for identifying SSI of POP. A dataset of ~1.8 million commercially available molecules was initially reduced to less than 12,000 which were screened by these models to a final set of 20 molecules which were sent for experimental validation (five random molecules were tested for comparison). Two molecules out of these 20, one with a high score in the ISE model, the other successful in the pharmacophore model, were confirmed by in vitro measurements. One is a competitive inhibitor of Ang-III (increases its K_m_), but non-competitive towards TRH (decreases its V_max_).

## Author summary

Many proteins are enzymes—"catalytic machines" performing chemical reactions on "substrates"–which may be small or large molecules. Evolution optimized the speed of enzyme reactions, but mutations or excessive enzyme production could lead to non-controlled, accelerated activity, which must be blocked to avoid a product that promotes disease. Many inhibitors of enzymatic activity became drugs which can block the production of the aberrant product, due to blocking the enzymatic "machinery", the amino acids involved in catalysis. Most enzymes have several substrates and so, those other substrates are blocked too. Those may be vital to the well-being of cells and life and total inhibition is prone to cause serious side effects. It is therefore essential to solve the need for inhibition of a single substrate without inhibiting others. We have thus developed computational methods to block specifically the "culprit" substrate while allowing the enzyme machine to act on other substrates. By applying these computational methods, we predicted candidates for inhibiting one out of two substrates ("substrate selective inhibition") of a well-known enzyme reaction. In collaboration with a research group that excels in studying that specific enzyme (prolyl oligopeptidase) we found that two candidates out of a set of twenty that we picked out of 1.8 million molecules by filtering through computer models—are indeed selective to one substrate vis-a-vis the other (five random molecules were tested for comparison). This may be the first example of a computational method leading to substrate selective inhibitor drugs which could avoid side effects.

## Introduction

Inhibitors of excess activities of proteins, mainly of enzymes, form a major group of clinical drugs. Unfortunately, most of these therapeutics cause side effects. The common explanation of side effects is that drugs interact with more than a single targeted protein due to similarity among enzyme families. Thus, effective blocking of an enzyme's active site might result in interfering with activities of other enzymes which are essential to the balance and viability of the biological system (called "off targets" or "anti-targets"). Due to the increase in the understanding of the commonalities in enzyme mechanisms and of enzyme "families", assay panels of proteins have become available in order to examine the selectivity of candidate inhibitor drugs. For example, see [1].

A different issue of selectivity came recently into public attention, as Semagacestat, a γ-Secretase effective inhibitor, failed in advanced clinical studies for treating Alzheimer's disease (AD). Semagacestat prevents the final cleavage of the amyloid precursor protein to beta amyloids which form toxic aggregates and are associated with AD. As it blocks the catalytic site of that aspartic protease, it prevents the cleavage of other substrates, mainly the vital Notch receptor, thus leading to toxic outcome [2]. Insulin-degrading enzyme (IDE) is another example of the demand for substrate selective inhibition [3]. Angiotensin-Converting Enzyme (ACE) cleaves many substrates such as Angiotensin-I (Ang-I), bradykinin, and others. ACE inhibitors were designed to block the cleavage of Ang-I to Ang-II, a major pressor, but prevent the cleavage of other substrates [4] due to blocking the catalytic residues.

Many if not most enzymes have multiple substrates, of which only a single one might be associated with disease, due to aberrant enzyme activity. Blocking the catalytic machinery then results in blocking all its substrates in addition to blocking the harmful one.

A solution to these limitations could be realized by applying the concept of "substrate selective inhibition" (SSI): a drug that should inhibit only a single substrate. That could be achieved by blocking that substrate's binding site on the target enzyme, without affecting other substrates or by blocking that substrate before it reaches the enzyme. Blocking a single substrate at the enzyme's binding site is based on the assumption (and hope) that there may be some part of the enzyme to which that substrate binds but is not shared by others of its substrates.

SSI's have been discovered by chance in some cases: small molecules were found as SSIs of Monoamine-Oxidase (MAO) [5], Cyclooxygenase-2 (COX-2) [6–8], and Phosphoinositide-dependent protein kinase 1 (PDK1) [9]; peptides were found to interact with the exosite of Dipeptidyl Peptidase 4 (DPP4) [10], and IDE [11] thus distinguishing between long and short substrates; An antibody was found as SSI of Pregnancy-associated protein A (PAPP-A) [12]. Novinec et al. discovered an allosteric inhibitor of Cathepsin K with different effects on two of the substrates (calf-skin collagen and azocasein) [13].

While serendipity in discovering SSIs may prove successful in some cases, our interest is in rational design/discovery of SSI, performed in silico, which could hopefully be more generalized. As a first attempt for such discovery, we focused on a candidate enzyme which binds and cleaves two different substrates at the same catalytic site. We focused on a serine protease, as the "pockets" of proteases around the catalytic sites are defined with respect to residues of peptide or of protein substrates [14].

Prolyl Oligopeptidase (POP) (E.C 3.4.21.26) cleaves short peptide hormones or neuropeptides (< 30 amino acids) between Pro and other amino acids [15], including: Substance P, Thyrotropin releasing hormone (TRH), Gonadotropin releasing hormone, Arginine-vasopressin, Angiotensins (I-IV), Bradykinin, Oxytocin, β-endorphin, Neurotensin, α-melanocyte-stimulating hormone and others [16]. POP activity has been associated with neurodegenerative diseases, and psychiatric disorders [16], as well as linked to blood pressure regulation [15]. It is easy to imagine how inhibition of catalytic residues of such an enzyme might lead to severe side effects.

POP has a typical catalytic triad of Ser554-His680-Asp641 but an unusual oxyanion hole ([17], see S1 Fig). Compared to other proteases, POP lacks pockets S3'-S4' [18], and interactions beyond the S3 pocket are weak [16]. Therefore, substrates are cleaved at S1-S1' while none of their residues may be bound further than S2'. Two of these substrates are Thyrotropin releasing hormone (TRH) and Angiotensin-III (Ang-III). Table 1 presents the residues of both for which K_m_ values were previously determined [19], with Ang-III being more strongly bound than TRH (0.6 μM vs. 98 μM, respectively).

**Table 1:** Two different substrates of Prolyl Oligopeptidase (POP).

POP pocket	Beyond S3	S3	S2	S1	S1’	S2’
**Substrate**	**P6-P5-P4**	**P3**	**P2**	**P1**	**P1’**	
**Ang-III**	R-V-Y	I	H	P	F	
**TRH**		pQ	H	P	NH_2_	

*pQ: pyroGlu

As both substrates (Ang-III and TRH) are cleaved at the same site, we questioned if it would be possible to: 1) design or discover by computational methods an inhibitor which does not interact with S1-S1', thus not blocking all possible substrates and/or 2) design or discover an inhibitor that will affect those two substrates differently.

As we mentioned above, a few discoveries of small molecules [5–9] and proteins [10–12] as SSI were published. We suggest here computational approaches to discover SSIs and present the successful outcome.

## Results

**Fig 1:**
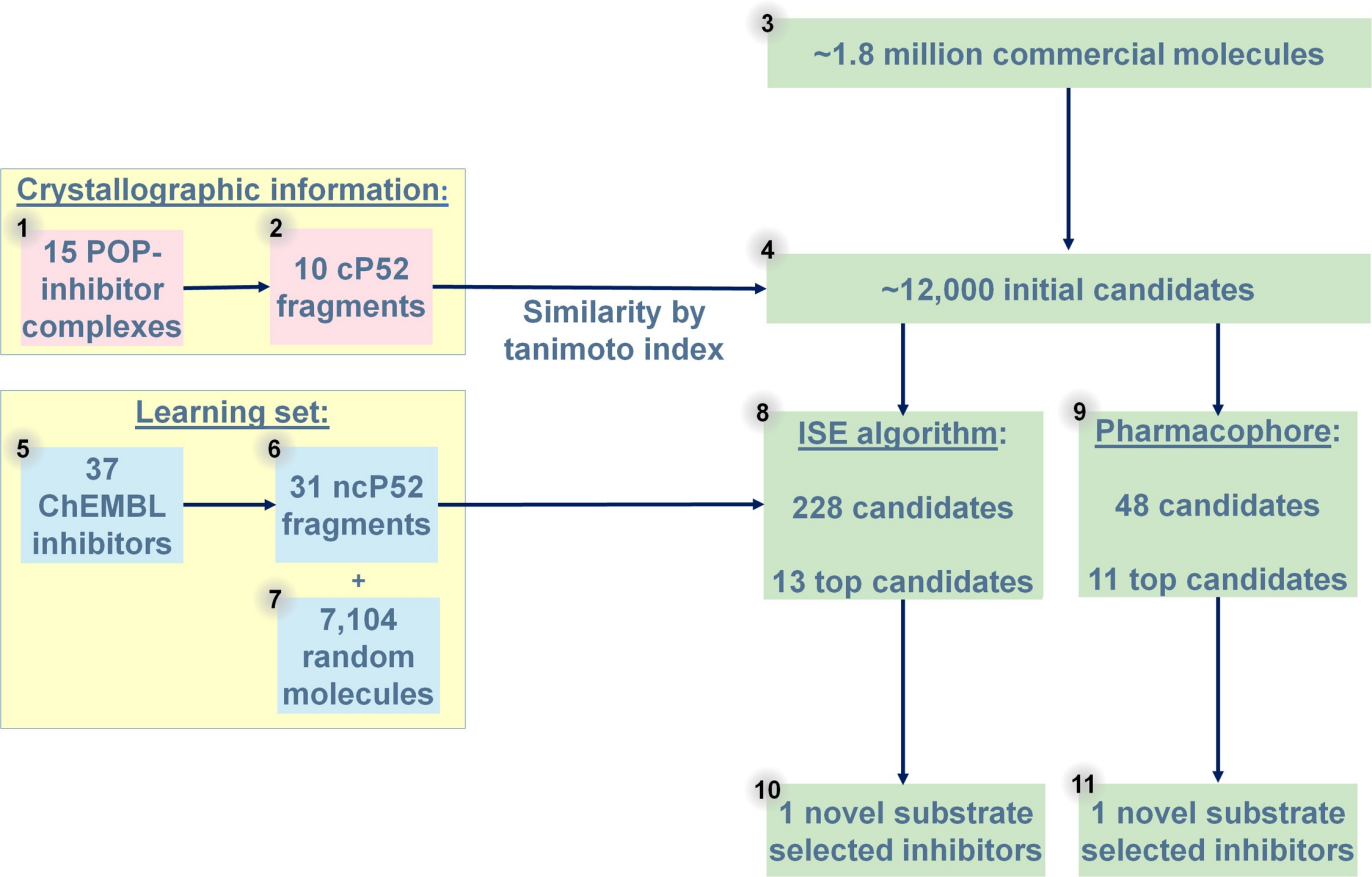
The process of filtering molecules for discovering SSI candidates. ~1.8 million molecules (3) were reduced to ~12,000 (4) by their Tanimoto similarity to fragments that are parallel to P5-P2 positions of the substrate (1+2, fragments from the full inhibitors, pink squares; and S2 Fig). Following the filtering based on ISE (8) and Pharmacophore (9) models, 20 molecules were selected by consensus prediction of solubility (12 from ISE, 8 from pharmacophore). ISE classification model was constructed based on (5+6) 31 fragments of POP inhibitors (from in vitro studies) against 7104 (7) randoms. Finally, two novel substrate selective inhibitors (SSIs) were discovered (10+11).

The process of SSI discovery starting from a huge commercially available library is presented in Fig 1.

### Superimposing crystal structures for retaining partial molecular inhibitor fragments

Having a crystal structure of an enzyme-substrate complex is possible in case a catalytic active residue has been mutated (as is this case of PDB structure 1E8N used here) or in case of a “slow substrate”. In case of a mutation, it is not obvious that the substrate resides in exactly the same position as it would have been in the native enzyme, which is not amenable to crystal structure analysis due to the fast reaction. However, a POP structure with a catalytic residue mutation S554A is the starting point for all our current study of POP.

**Fig 2:**
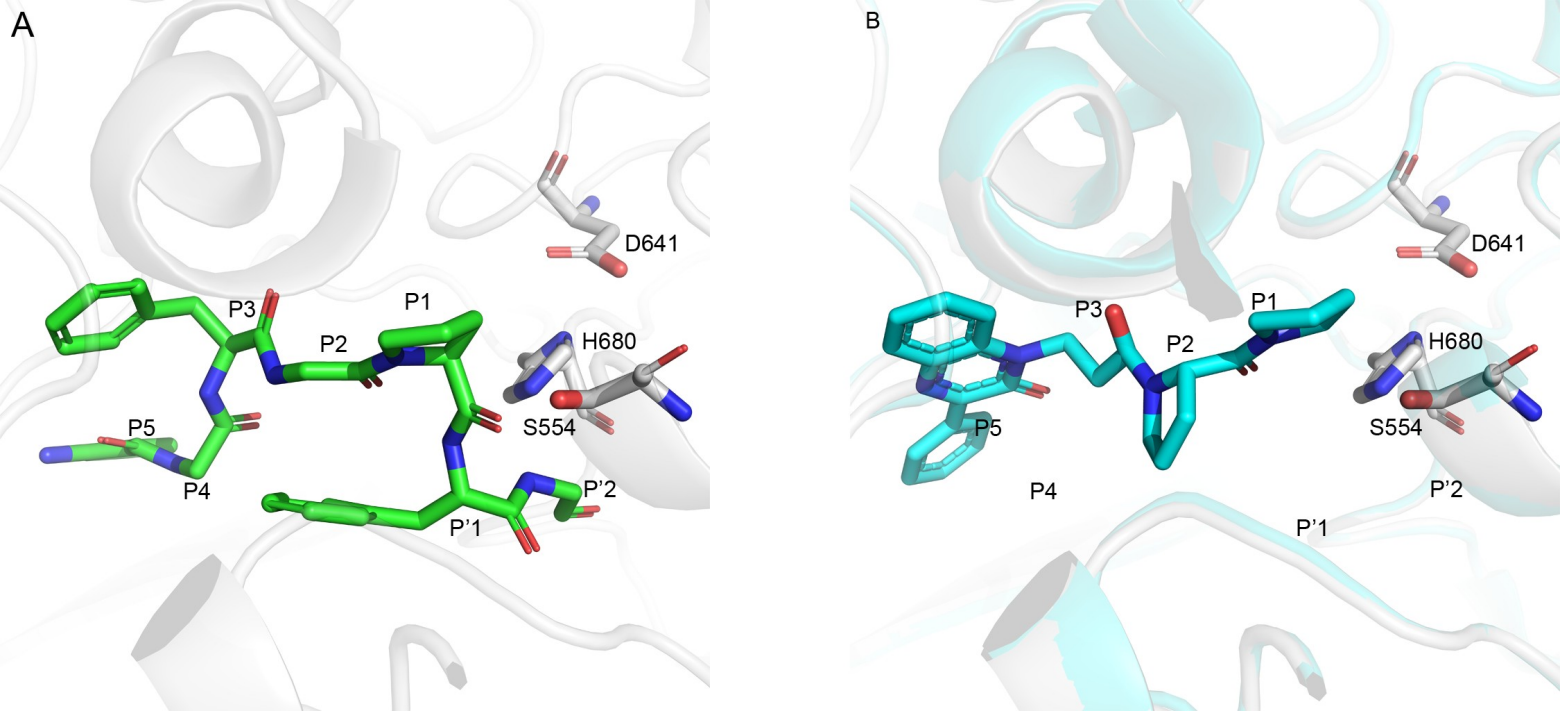
Substrate and inhibitor positions in the active site of POP. A) The substrate from structure 1E8N with Ala554 replaced by Ser so as to present its ability to attack the carbonyl of P1 for cleaving P1-P1'. B) An inhibitor positioned at the active site while its P1-P1' positions have been erased. Substrate and inhibitor are presented as green and cyan sticks, respectively and enzyme elements are in pale gray.

To avoid the blocking of peptide cleavage, we begin by superimposing the crystal structure of the inhibitor complex over the structure of the complex with the substrate. That, in order to identify which inhibitor fragments are equivalent to P1-P1' of the substrate cleavage positions, that reside in the protein pockets S1-S1'. Those inhibitor fragments should be rejected so as to supply partial structures that do not intervene directly with the cleavage. Other molecular parts between S1-S1' and the N-terminal are retained for subsequent modeling steps. To identify those inhibitor fragments, we superimposed the single POP-substrate complex (1E8N) over a set of 15 POP-inhibitor complexes. In Fig 2A we present the original substrate position next to the catalytic triad of POP (we "back-mutated" Ala554 to Ser) while Fig 2B shows the computer—aligned conformation of an inhibitor.

Most of the published complexes of POP-inhibitors are of Wild Boar (*Sus Scrofa*), and only one is a structure with a human sequence, with high sequence identity of 97.2% (690/710), and similarity of 98.9% (702/710) to the *Sus Scrofa* sequence. Pocket identification is also robust due to the high similarity in atomic positions, with RMSD of 0.21–0.37 Å between 1E8N and the other complexes (Average 0.29 Å, standard deviation of 0.04 Å). Detailed codes and main chain RMSD values are presented in S1 Table.

In all the complexes, molecular fragments analogous to the P1-P1' positions (all are similar to Proline) have been removed. Chemical structures of these inhibitors and the resulting set that we named "cP52" (for crystal positions P5 to P2) are shown in S2 Fig. Once compared to each other, only 10 unique cP52 fragments remain out of those 15.

### Producing the set of our candidate molecules

A commercial database, (Enamine,1.8 million molecules) [20] was screened against the 10 cP52 fragments to identify molecules that are most similar (by Tanimoto Coefficient (TC) [21]) to those fragments. We found 11,713 molecules which have TC of at least 0.6 to one or more of the cP52 fragments. Those were picked as our initial candidate SSIs and are the set from which we chose our 20 final candidates for experimental validation.

We do not have enough resources to test all the 11,713 candidate SSIs in vitro, thus we constructed two different models for subsequently picking a smaller number of potential candidates. The steps of model building are described in the next sections.

### Expanding the set of P5-P2 fragments from in vitro inhibition

We need to expand the set of fragments due to requirements of our classification algorithm, Iterative Stochastic Elimination (ISE), which needs at least a few dozens of "active" molecules for producing a model. We therefore picked from ChEMBL database [22] 174 known inhibitors from in vitro studies with IC_**50**_ or K_**i**_ values up to a maximum of 50 nM. By requiring a diversity of TC < 0.7 for all pairs among the 174, we remained with 37 inhibitors (S2C Fig) and following the cleavage, in each molecule, of the assumed P1-P1' parts, we remain with 31 fragments that we call "non-crystallographic positions P5 to P2" (ncP52). That is the set used as "positives" for our classification models (S2D Fig).

The parts that were "chopped off" the 37 in vitro molecules are the proline like rings of pyrrolidine or piperidine. Substitutions beyond that ring (relative to the correct orientation) were removed.

To avoid bias in combining the two sets, we compared the 31 ncP52 fragments to the 10 cP52. S3 Fig presents the matrix of Tanimoto values between the sets: lines are for the ncP52 set of 31, and columns present the cP52 set from crystal structures. Among 10 cP52 fragments, four are found in the ncP52 set. The six remaining cP52 will be used as an "external" test set. Out of the set of 11,713 initial candidate SSIs based on cP52, 7,900 were picked by similarity to six unique fragments of the 10 cP52. By constructing the ncP52 set: in 23 out of the 31 ncP52 fragments (70%) the maximum similarity to cP52 is TC < 0.7.

### The learning sets

We wish to create models that enable to discover active molecules by screening a very large number of candidates through these models. In real life, such as in High Throughput Screening (HTS), only a very small fraction of huge molecular libraries is discovered to have a specific activity [23]. Therefore, to produce a classification model with our computational approach while mimicking HTS by virtual screening (VS), we need to construct a learning set by diluting active molecules (such as our ncP52 sets of partial inhibitors) with a huge number of inactive molecules (in our case, non-inhibitors). But as we have no access to data of failed POP inhibition studies, we picked some 10,000 molecules by random choice out of the 1.8 million molecules proposed by Enamine, assuming that the chance to pick an inhibitor of POP among those random molecules is extremely low. Those molecules are our class of inactives.

Subsequently, we used applicability domain requirements [24] based on properties of the set of 174 original inhibitors to reduce the initial ten thousand molecules, to a final set of 7,104 inactives for learning. Lipinski rule variables [25] (but not values) were used for the applicability domain based on their average minus/plus standard deviations (-2σ to 2σ) (see S2 Table).

The final learning set, including 31 ncP52 (the actives or "positives") and 7,104 random molecules as inactives (or "negatives") was used 1) to validate a pharmacophore model and 2) to construct a filters' model by our in-house "Iterative Stochastic Elimination" (ISE) algorithm. In addition, two external test sets were examined by these 2 models– 37 original (full) inhibitors (the basis for the ncP52 set) and 6 out of the cP52 set from crystallized ligands. The original 37 inhibitors should be characterized as negatives by our models while the cP52 set should be identified as positives.

### Pharmacophore model results

The details of three different "automatic" models and one visual inspection model are presented in the Materials and Methods section. S3 Table presents the coordinates and the types of the pharmacophore features based on the structure 1E8N. Those were used to examine the ability to separate between the 31 ncP52 fragments (expected to be positive) and random molecules, as well as the full inhibitors (both should be negatives). H-bonds are given as vectors with coordinates of origin and “target” (tip), each. Hydrophobic and excluded volume are given the coordinates of their center, with standard program radii.

Each of three sets: 31 fragments of ncP52, 7,104 random molecules and 37 original inhibitors was tested by all four methods. The criterion for evaluation of the results is True vs. False positives. For ncP52 fragments and the random molecules the TP/FP in the four pharmacophore tests were: 7 (out of 31) vs. 2 (out of 7104), 7 vs. 2, 8 vs. 1 and 8 vs. 2. The enrichment is thus very large. The full inhibitors (from which ncP52 are derived) did much worse than ncP52 with respect to the random molecules: 2 (out of 37) vs. 2 (out of 7104), 2 vs. 2, 3 vs. 1 and 3 vs. 2. The pharmacophore tests thus prefer the ncP52 fragments. For more details, see S4 and S5 Tables.

We require top molecules to pass successfully all 4 pharmacophore methods. Out of 31 fragments in the ncP52 set, 6 passed well in all 4. Only one of the full inhibitors (out of 37) passed all 4, and none of the random 7,104 molecules passed all 4.

We screened the 11,713 candidate SSIs (full molecules) requiring the same criteria. Eleven molecules (our "top candidates" in the pharmacophore modeling) were positive by all 4 methods, and further 37 were successful in at least one method (see S4 and S5 Tables)

### Iterative Stochastic Elimination (ISE) results

Using ISE [26–30], a five-fold model (see Materials and Methods) of the ncP52 set (31 fragments) vs. the random set (7104) was constructed. The AUC was 0.97 (see ROC curve in S4A Fig) [31]. The top filter has a MCC (Matthews Correlation Coefficient) of 0.95 (none of the top filters in the different five folds has MCC < 0.9). We tested the stability of these results by screening the full inhibitors through this mode. The AUC is only 0.59 (S4B Fig). Thus, the model distinguishes well between ncP52 fragments and random molecules while it cannot distinguish between the full inhibitors and random molecules.

**Fig 3:**
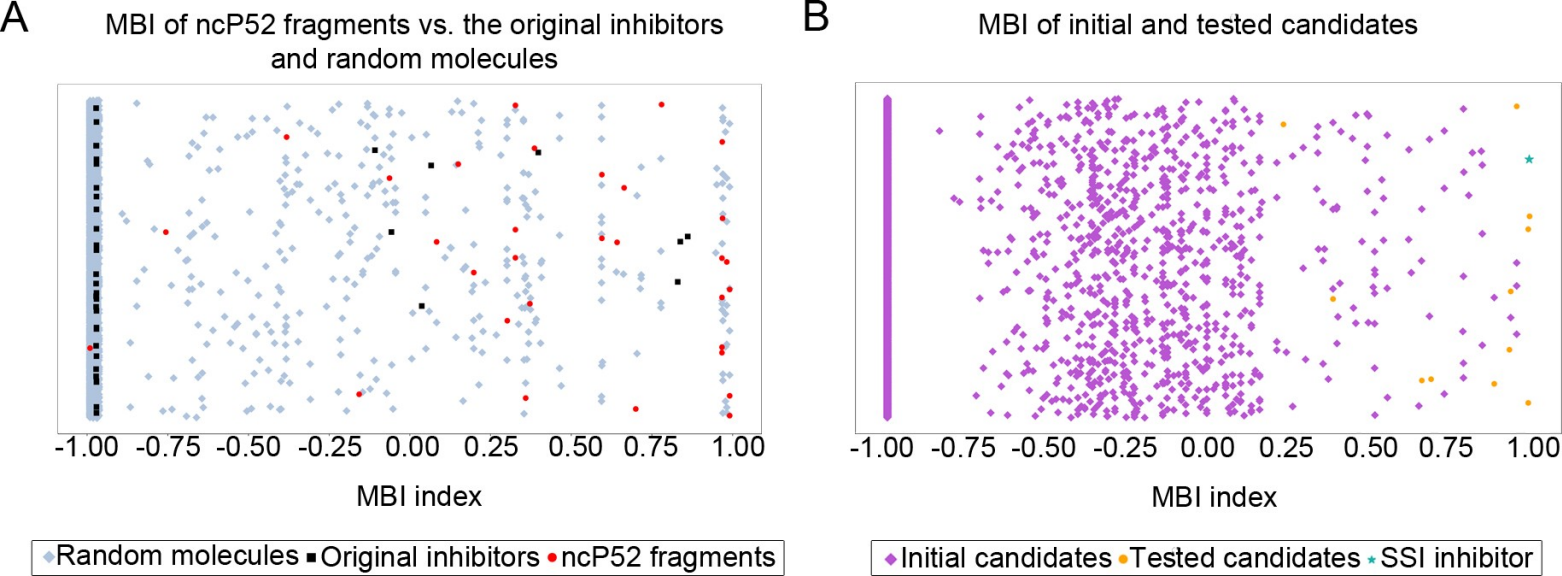
Distribution of different sets according to the MBI index. A) Distribution of the ncP52 set (in red), the original inhibitors (in black) and the random molecules (in blue). B) Distribution of the virtually screened 11,713 initial candidate SSIs (in purple). Orange circles represent the molecules that were sent for in-vitro tests (the hit by ISE algorithm is presented as a cyan star).

Fig 3 presents scatter plots of three sets according to their Molecular Bioactivity Indexes (MBI, see Eq 2 in Materials and Methods) which are the result of scoring each molecule by all the filters of the model: 3A) the results for ncP52 fragments are shown in red, black squares represent results of the 37 full inhibitors and blue diamonds are for random molecules. S6 Table presents the numbers of ncP52 fragments and of random molecules that have values above different cutoffs of MBI–those are true Positive (TP) if they are from the ncP52 fragments and are False Positives (FP) if they are from random molecules. Values of (TP/FP) above specific MBI indexes are used as a major criterion for decision on subsequent picking of candidates from virtual screening. For MBI > 0.85 we found TP/FP of 10/33 (10 out of 31 ncP52 vs. 33 randoms out of 7,104), which may be interpreted as an expectation to find 10 actives among 43 (10 + 33) molecules with MBI > 0.85 that may be sent for experimental evaluation. Of course, that number could be smaller, so that to find at least 5 actives we may send about 22 molecules for experimental evaluation. Of the 37 full inhibitors, however, only one (!) was found to have a MBI above 0.85. Thus, the ISE model distinguishes well between total inhibitors and fragments which are SSI candidates. Based on that result from the ISE model, we decide to pick top candidates of the subsequent virtual screening by that model only if their MBI < 0.85.

To further validate the ISE model, external sets mentioned above were screened. The six unique cP52 fragments (fragments from POP inhibitors in crystal structures) that were found to be dissimilar to the ncP52 set (see S3 Fig), and a random set of 5,450 molecules from Enamine were given MBI values by the model's filters. The random set was picked by "applicability domain" defined with properties of the 10 crystallographic inhibitors. While the unique cP52 fragments have MBI > 0 (three of them have very high values of 0.895, 0.914 and 0.933), the original crystallography inhibitors (from which the cP52 fragments are derived) have MBI < -0.971 (see S7 Table). Only 14 of the 5,450 random molecules have MBI values above 0.85, so the TP/FP is 3/14 (S6 Table) above that MBI value. As these external set evaluations reflect the ability of the model to be validated in the "real world", this TP/FP suggests that for any 17 molecules (3+14) sent for experimental validation, 3 could be found to be SSI. The pharmacophore model could not distinguish that well between the six cP52 fragments and their original inhibitors.

### Screening and picking top candidates for experimental validation

The decision to pick candidates from virtual screening is based on the analysis of TP/FP values at different cutoff values of the indexes in any model. We screened the 11,713 initial candidates by the ISE model. Only 947 of those have MBI > -0.9; 228 of those are found to have a positive MBI > 0.0 (for details–see S6 Table). But only 13 molecules have MBI > 0.85 and were thus defined top candidates. Fig 3B presents a distribution of this set according to each MBI.

Solubility of molecules is a crucial property for in vitro enzyme kinetics, but rather than measuring solubility of many candidates, we compute those values with different algorithms. Five such algorithms were used to calculate cLogS for the candidates, with averages and standard deviations calculated for each molecule. An average of cLogS > -3.5 and a small (< 1.5) standard deviation is a necessary condition for defining a molecule as a candidate to be sent for experimental testing.

Picking molecules for in vitro tests is limited by the capabilities of the experimental lab. We decided to pick our candidates by both pharmacophore and ISE in order to hopefully learn about the validity of the methods and to increase the chance for discovery. Twenty SSI candidates were finally sent for in vitro tests. Five additional molecules picked randomly were added for validation. All 25 molecules fall into the following categories: 1) Pharmacophore candidates– 8 molecules that succeeded at least in one of the pharmacophore approaches; among them, 2 are top pharmacophore candidates, as they succeeded in all 4 methods; 2) ISE candidates– 12 molecules, out of which 8 are top candidates with MBI > 0.85 and 4 have lower values (0.2 < MBI < 0.85); 3) Three molecules were picked among the worst computed MBI values (MBI < -0.97) by structural similarity to the cP52 fragments (these molecules also failed in the pharmacophore tests); that, in order to examine if structural similarity is or is not a sufficient condition for successful discovery of SSI; 4) Two molecules were picked randomly among the ~1.8 million molecules of Enamine (we validated that these molecules have MBI < -0.97 in the ISE model, as well as failed according to all of the pharmacophore methods); that, in order to validate that POP pocket is not promiscuous and could accommodate random molecules. S5 Fig presents the structures of the molecules that were sent to in-vitro tests.

### Experimental testing and analysis of the results

Preparation of recombinant human POP (rhPOP) is described in the Experimental procedures. The obtained rhPOP showed no enzyme activity other than the substrate specificity of POP for Ang-III (Arg-Val-Tyr-Ile-His-Pro-Phe) and TRH (pGlu-His-Pro-NH2). The chromatograms obtained by analysing the reaction mixture of rhPOP with Ang-III or TRH without inhibitor by RP-HPLC are shown in S6 Fig. In S6A, the peak at RT 11.0 min was that of Phe at the C terminus of Ang-III. In addition, it was confirmed by the protein sequencer (Procise 491HT; Applied Biosystems) that the peak at RT 15.8 min was that of Ang-III fragment (Arg-Val-Tyr-Ile-His-Pro). In S6B Fig, the peak at RT 17.0 min was determined to be that of TRH fragment (pGlu-His-Pro) by MALDI-TOF (microflex; Bruker) MS analysis (see S7 Fig).

**Fig 4:**
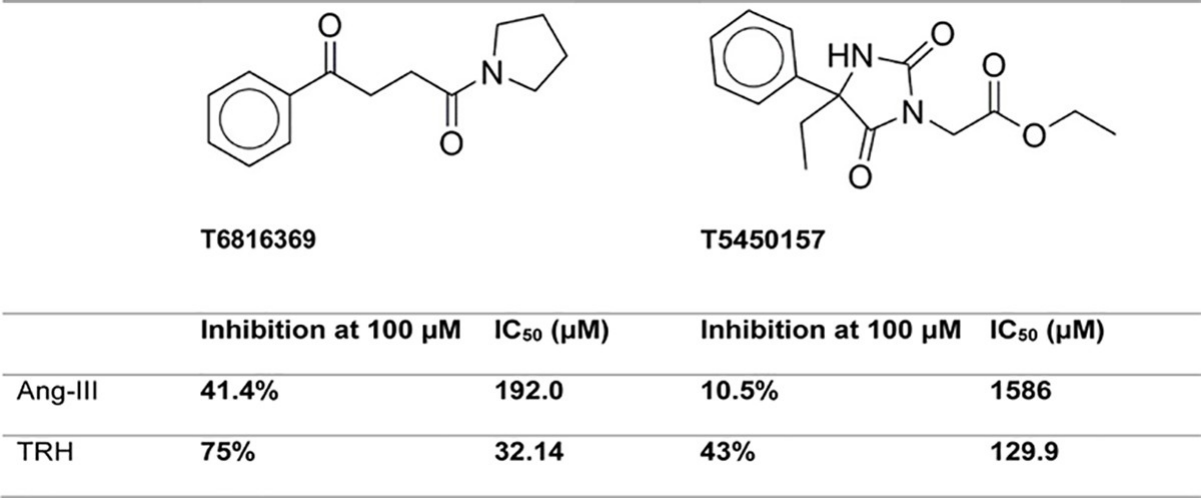
Reduction of POP catalytic action by the two novel inhibitors, shown at the top of the figure.

First, the effect of 100 μM of inhibitor on the POP catalytic action toward Ang-III and TRH was investigated for 20 candidates and 5 decoys for confirmation (see S8 Table). Two of the candidates were found to affect differently the two substrates (Ang-III and TRH) of POP (Fig 4). In presence of these inhibitors, catalytic activity was inhibited between 10–75%. One of the two, T6816369, succeeded in all 4 "structure based" pharmacophore tests and the other, T5450157, was successful in the "ligand based" ISE candidate set, with the 2nd highest MBI of 0.971.

**Fig 5:**
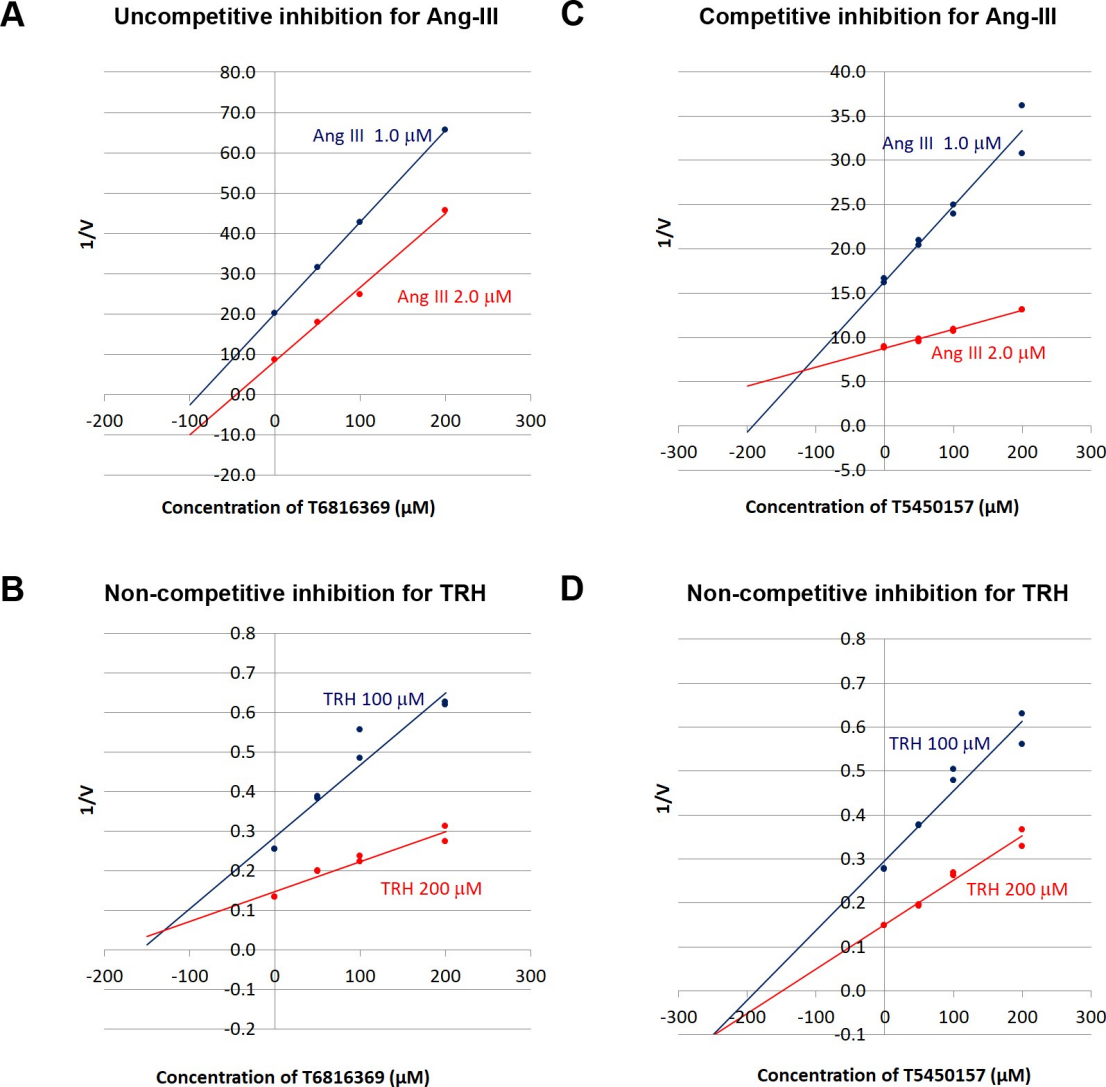
**Dixon plots of:** A) Ang-III cleavage in presence of T6816369; B) TRH cleavage in presence of T6816369; C) Ang-III cleavage in presence of T5450157; D) TRH cleavage in presence of T5450157.

Values of K_m_ and V_max_ were determined for the two inhibitors. Fig 5 and Table 2 present respectively the Dixon plots and the effects of these two inhibitors on the K_m_ and V_max_ values for the Ang-III and TRH cleavages. T5450157 displays a competitive inhibitory action for the longer Ang-III substrate. K_m_ for Ang-III is increased (1.617/0.936 = 1.73) in presence of T5450157, while V_max_ (0.1118/0.1078 = 1.04) is unchanged. In contrast, in presence of TRH, only the V_max_ value are decreased (12.46/19.08 = 0.65), but not the K_m_ (718.4/690 = 1.04), and therefore this inhibitor is non-competitive towards TRH [32,33].

**Table 2:** Values of K_m_ and V_max_ extracted from Michaelis-Menten equations.

Substrate	Ang-III		TRH		TRH/Ang-III–ratio	
Candidate	K_m_ (μM)	V_max_ (μM/min)	K_m_ (μM)	V_max_ (μM/min)	K_m_	V_max_
[1] Control	0.94	0.11	690.0	19.08	716.51	176.99
[2] T6816369	1.20	0.06	1168	9.191	974.96	145.66
[3] T5450157	1.617	0.12	718.4	12.46	444.28	111.45
[2] / [1]	1.28	0.59	1.69	0.48		
[3] / [1]	1.73	1.04	1.04	0.65		

In the case of T6816369, K_m_ value was increased for both Ang-III (1.198/0.936 = 1.28) and for TRH (1168/690 = 1.69). V_max_ values for that inhibitor have been modified substantially, to two-thirds of the control with Ang-III (0.0631/0.1078 = 0.59) and to half of the control with TRH (9.191/19.08 = 0.48). From the Dixon plots of this candidate, it seems that it is an uncompetitive inhibitor for the Ang-III cleavage, but non-competitive inhibition for the TRH cleavage.

Finally, we measured IC_50_ for the two inhibitors (Fig 4). T6816369 has IC_50_ values of 32.14 μM (Std. error 1.07) and 192.0 μM (Std. error 2.42) for TRH and Ang-III, respectively. T5450157 is a weaker inhibitor, with an IC_50_ of 129.9 μM (Std. error 6.78) and 1586 μM (Std. error 92.5) for TRH and Ang-III, respectively.

These differences in inhibition potential vis-à-vis two substrates of POP confirm the ability of our computational methods to produce inhibitors that distinguish between substrates of a specific enzyme, POP in the present case.

### Docking the candidates to the POP structure

By docking the experimentally successful candidates we wish to examine the relevance of mechanistic assumptions and their relation to the experimental results. The experiments suggest that T5450157 acts as a competitive inhibitor for the cleavage of the longer substrate (Ang-III), and acts as non-competitive inhibitor for the shorter one (TRH). These experimental in vitro findings are according to our expectations from the design, suggesting that the SSI binds outside of the catalytic site. Indeed, we assume that T5450157 interacts with the S5-S4 sub-pockets. If so, it blocks the binding of the longer substrate that needs these sub-pockets (thus, competitive inhibition), but does not block the binding of the shorter TRH substrate that occupies the S3-S1’ sub-pockets only (and so its inhibition is non-competitive) while it can still interact with TRH and affect its turnover.

We examined that possibility by docking the 20 candidates to the area of the S5-S4 sub-pockets. By cleaving (computationally) the P5-P4 positions of the crystallographic substrate (PDB ID: 1E8N), the P2’-P3 positions are thus presented as a possible short substrate (as TRH would be), while the P5-P4 positions serve as a "ligand reference", to define the position of the docking pocket.

**Fig 6:**
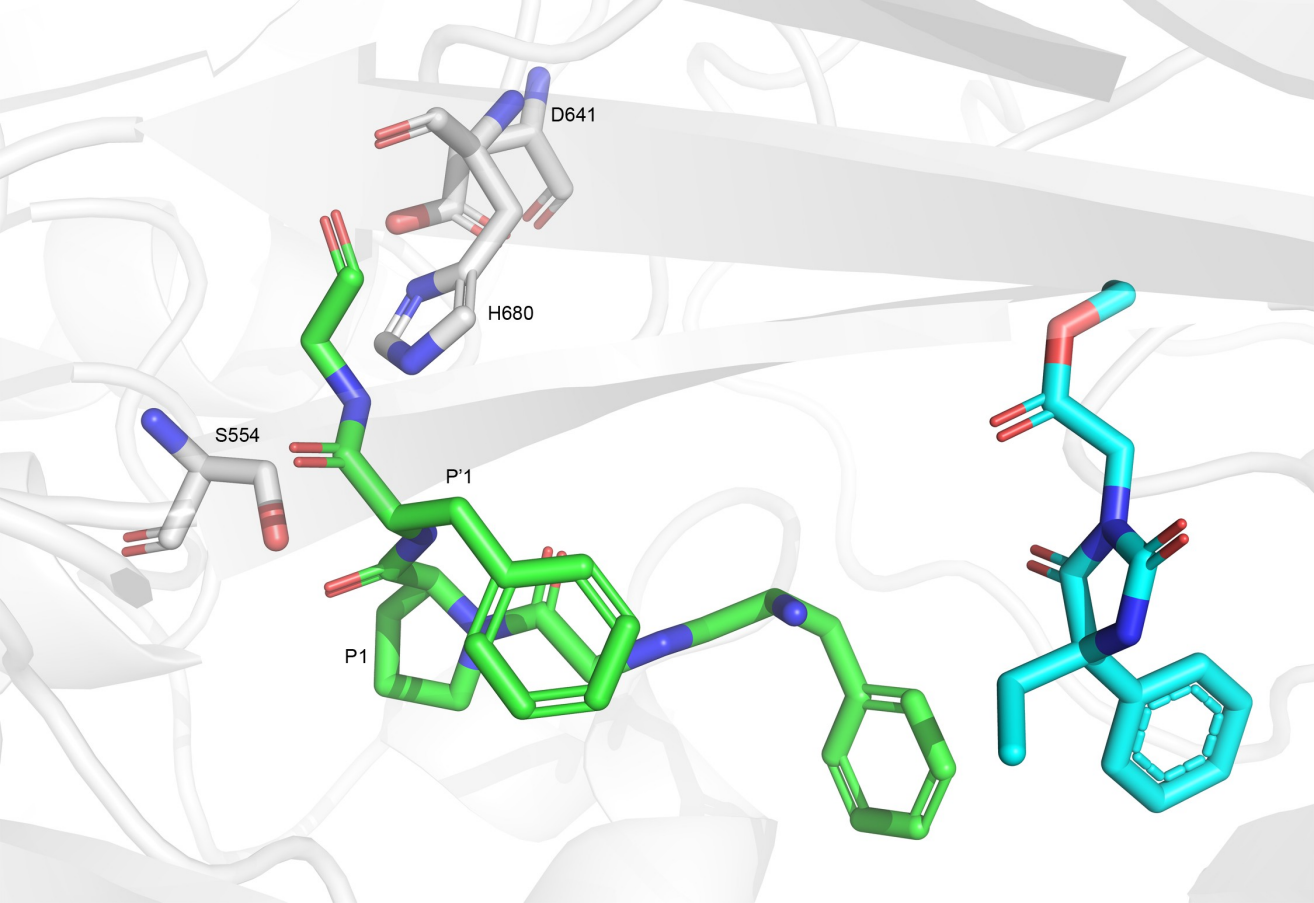
Docking pose of the competitive SSI. Carbon atoms of the catalytic residues are presented in white; the substrate's carbons are green and relevant P positions are indicated. The SSI carbons are presented in cyan. T5450157 is the competitive inhibitor of Ang-III and is shown occupying closely the P3-P4 positions of Ang-III. The inhibitor is expected to affect any longer substrate.

Rigid docking was performed by Glide (Schrödinger) [34], with the SP (standard precision) algorithm. Out of the 20 candidates, which were allowed to dock in up to 50 conformations each, only 7 candidates have at least one conformation at the S4-S5 position in the protein, indicating that most of our candidates could not dock well. T5450157, the novel SSI, has 10 such successful conformations (out of 286 of all the seven candidates), but a conformation of T5450157 (Fig 6) was ranked as the top of all 286 by Glide score. The Glide score for this conformation was -5.15 (kcal/mol), while most conformation of other molecules appear at higher energies (i.e., the third best has an energy > -4.75). It should be noted that 5 of the top 10 scored conformations were of T5450157, better than any other candidate.

## Discussion

Inhibiting enzymes by blocking their catalytic residues has been the standard method for design/discovery of enzyme inhibitor drug candidates, which block the processing of all enzyme’s substrates. This collides with the central premise of our approach, which attempts to avoid total blockade, resulting in unwanted inhibitions of substrates that require cleavage for maintaining vital cellular function. Avoiding interactions with catalytic residues is central to our approach and was at the core of both methods that we used, pharmacophore and ISE. We finally found two inhibitors, one by each method.

One of the two in vitro SSIs, T5450157, is a competitive inhibitor of Ang-III (the longer substrate) and a non-competitive inhibitor of TRH. Fig 6 suggests the possible mechanisms for that difference. Our interpretation is that the competitive inhibition of Ang-III is due to blocking the area beyond S3 (towards the N-terminal) required for Ang-III binding, while TRH does not need these binding sites and can still bind to POP, thus causing non-competitive inhibition. Competitive inhibition is reflected by an increase in the value of K_m_, thus reflecting less binding affinity of Ang-III with that inhibitor. K_m_ however is not increased for TRH while inhibition by T5450157 takes place.

### Significance of the results

Is the increase of K_m_ (for competitive inhibition of Ang-III by T5450157) significant? The K’_m_/K_m_ ratio for inhibiting the cleavage of Ang-III by T5450157 is 1.617 μM/0.936 μM = 1.73 (Table 2). This ratio is similar to some others in the literature. For example, Bradykinin inhibits thrombin, a serine protease. The change in K_m_ of the chromogenic substrate S-2238 is 10 μM/5.76 μM = 1.74 (for human thrombin) or 11.5 μM/7.11 μM = 1.62 (for bovine thrombin) [35]. Another example is of Diosmetin inhibiting the biotransformation of Diclofenac by Cytochrome P450 2C9. Without the inhibitor, the K_m_ value is 9.5 μM; with Diosmetin concentrations of 1 μM, 2.5 μM and 4 μM, the K_m_ values are 13.85 μM, 21.52 μM and 23.95 μM, respectively) [36]. Thus, the ratio between the K_m_ values, depending on concentration, was 1.46–2.52. Another case is the inhibition of BACE-1 cleavage of "substrate-1" by binding fragments: the K_m_ of substrate-1 was increased due to inhibition from 4.97 μM without the inhibitor to 14.3 with inhibitor [37].

Our novel SSI is a small molecule (compared to Bradykinin, a nona-peptide), and thus it can make less interactions. Nevertheless, the ratios are similar. The novel SSI is not a strong inhibitor (IC_50_ of T5450157 for Ang-III is ~1586μM; bradikynin inhibiting bovine thrombin has Ki of ~270μM). However, this paper focuses on the issue of computational methods to discover SSI and not on the optimization of SSI potency.

Inhibition of Ang-III cleavage by T5450157 is in the mM range (IC50 = 1586 μM). Depending on doses, such concentration may not be relevant biologically. But this result is in line with our goal of inhibiting one molecule’s cleavage less than the other molecule’s cleavage which amounts to “substrate selective inhibition”.

Our measurements of K_m_ values for POP are different than what we find in the literature. While in the literature [19] the K_m_ values are 98 μM for TRH and 0.6 μM for Ang-III (and the ratio is 163), in our measurements (Table 2) the K_m_ values are 690 μM and 0.936 μM, respectively (and the ratio is 716.51). Thus, in the presence of T5450157 the K_m_ values are 718.4μM for TRH and 1.67μM for Ang-III, and the ratio is 444. This different ratio is expected as we designed competitive inhibition of the longer substrate only. But as an unexpected result, the shorter substrate, TRH, is inhibited in a non-competitive mode.

### A proof of concept for modeling SSI

How common are situations in which we wish to inhibit a single interaction of a protein which interacts with other proteins or peptides as its substrates? In the introduction we presented few cases of such enzymes. However, the problem is highly important as most of the activities of proteins depend on interactions with one another [38]. For example, the interaction between Calcineurin and NFAT is pathogenic, but interactions of Calcineurin with other “substrates” are essential [39]. Interactions between receptors and hormones are another area. For example, Denley et al. [40] suggested that inhibiting IGF-II (Insulin-like Growth Factor II) has a desirable effect for cancer therapy, but it seems that inhibition of other Insulin receptor (IR) signals affect glucose metabolism. Interacting with small hormones like IGF-II is challenging. However, in our approach, we can hope to design some substrate selective inhibitors of IR that will inhibit the interaction with IGF-II, but not with others (Insulin for example). We have started with POP, a protease, as proof of concept, as in proteases the subpockets are well defined. The potential extension of this approach to SSI is now open for examination on other proteins.

### Is that a "fragment based" discovery?

Our approach is totally different than the currently well known "fragment based" design. In that method, relatively small fragments that display binding affinity to a desirable protein site are either combined or extended in order to increase the binding affinity to what one expects from a "full" molecule [41]. We created our fragments from full molecules and eliminated molecular pieces that interfere with our designed goal. Also, our intention is to use fragments in order to construct models.

### Which technique provides better candidates?

The ISE ligand based model achieved a better result than the pharmacophore model. Based on our kinetic measurements and supported by docking, inhibitor T5450157, a result of ISE, fills the S4-S5 subpockets while inhibitor T6816369, based on the pharmacophore model, resides beyond the S4-S5 pockets. Another difference between ISE and pharmacophore models is in their scoring of the crystallographic original inhibitors compared to the scoring of fragments in both. The original inhibitors are not expected to score well compared to the fragments which are the basis of the models. S6 Table presents the difference in results between the full (original) inhibitors and the fragments, in their MBI scores of ISE. In contrast, as mentioned above, we have not found any difference between these sets according to the pharmacophore model. A possible explanation for the relative success of ISE compared to pharmacophore is that the latter is based on a crystal structure and is more "rigid" while the ISE model is based on a large set of fragments from structures that could attain different conformations. In that case, the ISE model allows more flexibility around the binding site.

### Enzyme inhibition SSI–what could be achieved?

Our original expectation was that it will be possible to block the longer substrate while allowing the shorter one to continue being processed. That, as a result of blocking only subpockets that interfere with the longer one, so that its inhibition would be much more prominent than inhibition of the shorter substrate. Our experimental results show the opposite: while we could expect that TRH would hardly be affected or not at all by blocking the S4-S5 pockets (competitively inhibiting Ang-III), it turns out that the shorter TRH substrate was inhibited more than the longer one, Ang-III (IC_50_ of Ang-III is 1586μM compared to 129.9μM for TRH). As Ang-III has much stronger affinity to POP than the affinity of TRH, that may be enough to explain that result.

We dealt only with a very simplified case of two substrates, albeit very different in size so that it may be possible, at least schematically, to block one or another. However, that depends also on the binding affinity of each: in our case, the longer peptide has a stronger binding affinity as expected. An inhibitor with greater affinity is required to reject Ang-III while an inhibitor with lesser affinity may be sufficient for TRH.

### Do we discover better than random?

Several methods could be used for discovering SSI. The most prominent one is probably massive docking limited by the specific requirements of inhibition. That may be an extremely lengthy process with unclear outcome. In our case, we limited our search for inhibiting one out of two substrates. Along the discovery process, we reduced the number of possible candidates to those that include appropriate fragments, and achieved that by "trimming" known inhibitors to the scaffolds that could serve the discovery. In the final step, we screened (by ISE and pharmacophore) only those ~12,000 molecules, a very small number out of the original ~1.8 million in the library, to reach the final candidates.

We discovered only 2 SSIs out of 20 that were sent for evaluation (the other five molecules were used as control, not being predicted by either ISE or pharmacophore). Is that a failure of the models? Should we have discovered more? We may tackle that question by using an example from "real world" screening. In HTS, it is expected (but not guaranteed) to find about 1 active molecule out of a thousand screened [23,30]. Therefore, to mimic that level of success in VS (Virtual Screening), a model of actives vs. assumed inactives should also be based on a 1 (true positive):1000 (true negatives) ratio. The ratio in our model is 37:7104, roughly ~5:1000. All these 37+7104 molecules are scored by the model's filters, so that at each score we can calculate the ratio of True to False Positives, TP/FP. For example, if we require that molecules screened by our model must have a minimal MBI of 0.85, we calculate the expectation value for that limit from the results of the model. We used MBI > 0.85 above which we find 10 fragments and 33 randoms, which suggests that out of 43 molecules picked from screening above MBI of 0.85, 10 molecules (~ 1 in 4) may be found to be SSI. But, as the ratio in the modeling was 5 times smaller (5:1000 instead of 1:1000) we should have expected to get about 10 to 33X5 = 165, meaning that we may find only 10 true actives if we send 175 molecules (165+10) for testing. We sent only 12, so that we could hardly expect even a single success. In fact, our success is even greater because among our 12 ISE candidates only 8 have MBI > 0.85 while four have MBI > 0.2, where the number of false positives is larger.

### Conclusions

We demonstrated the ability of computational approaches to discover substrate selective enzyme inhibitors (SSIs). Those and other computational methods could be extended to other enzymes and to protein interactions with small molecules or with peptides and proteins.

As much as this may be a first ever attempt to discover/design an SSI by computations alone, it is a limited one–both in the focus on a specific protease (which makes it easier to identify "pockets" and their substrate/inhibitor contents) and in the limited need to block one substrate while allowing just one other to be processed. In most cases, enzymes have more than two substrates, these substrates do not necessarily differ in size as TRH and Ang-III are, and the subsites are not as clearly defined. We benefited here also from a crystal structure with a small mutation at the active site (Ser to Ala) which we assumed to still reflect the correct substrate binding of the non-mutated POP. An even better starting point could be provided by an enzyme complex with a "slow substrate", so that there will be more valid substrate positions. The task of providing a single SSI while allowing the processing of a few other substrates is clearly more complicated but the initial clues to such construction have been presented here to some extent.

There are many other interactions for which it may be desired to halt a specific one or a few of them. Our approaches, with both a ligand based classification optimization method such as ISE and a structure related method such as pharmacophore may direct other researchers to extend these or other methods for providing better and more specific drug candidates.

## Materials and methods

### Computational procedures

#### Superimpose between the enzyme-substrate complex and enzyme-inhibitor complexes

POP complexes were collected by the following criteria: 1) the method is X-ray crystallography; 2) the resolution is < 3.0Å; 3) the structure is a complex of POP and an inhibitor. The complexes with inhibitors are superimposed over the complex with the substrate to identify commonalities. This is performed by Sybyl-X 2.0 [42]. Only the main chain atoms served for superimposing. The source of fourteen of those X‐ray complexes was from *Sus Scrofa*. In the case of a single human complex, we have aligned its sequence vs. the porcine sequence by Pairwise Sequence Alignment by EMBOSS Needle [43], and then we superimposed the complexes according to the alignment.

#### Curation of the various sets

Inhibitors from the crystal structures were extracted from the PDB entries (SMI format). For creating inhibitor fragments that could occupy pockets between S2 and the N-terminal, we introduced the full SMI code of each inhibitor into the ZINC database [44], removed the fragments that are in positions P1-P1’, and extracted the newly produced SMI codes. The same process was performed for the inhibitors that were extracted from the ChEMBL database [22], but since we do not have the crystallographic positions, we decided to remove the proline-like rings (For details see S2 Fig). We defined the fragments from the crystallographic inhibitors cP52 and the non-crystallographic inhibitors are the set called ncP52. Sets of random molecules and initial candidate SSIs were extracted from Enamine Database [20] (~1.8 million molecules) according to applicability domain or Tanimoto Coefficient (TC), respectively. Random molecules were collected twice, once regarding the ncP52 Lipinski features as "applicability domain", and once regarding the cP52 Lipinski features.

Comparisons [21] of TC between the various sets and among molecules from the same set were calculated in OpenBabel (FP2 fingerprints) [45]. Highly similar molecules were eliminated to reduce bias. Out of a pair of highly similar molecules the one which has a larger sum of TCs with all other molecules was eliminated.

#### Construction of the pharmacophore models

For the complex of the POP-substrate (1E8N) [18] we used Sybyl-X 2 [42] to prepare it for the construction of a pharmacophore model. Water molecules were removed, and hydrogens were added to the whole protein and were geometry optimized. We transformed the mutated Ala-554 back to its original Ser-554 by adding oxygen to Cα and optimized the Ser–OH group in the field of the protein. Tripos Force Field was used with Gasteiger-Hückel charges, and a Dielectric Constant of 4. Minimization was performed by applying Steepest Descents followed by the Conjugate Gradient method, with 10,000 iterations in each minimization subject to a cutoff < 0.001 kcal / (mol*Å). The angle between the catalytic oxygen of Ser554, the unprotonated nitrogen of His680, and the carbonyl of TRH (P1 position) was maintained < 90° (82.8°).

Initially, a pharmacophore model was created with the full substrate in order to optimize the number of required features for distinguishing between "positives" (ncP52 set) and "negatives" (the random molecules). It includes 21 features of the following types: An H bond donor, four H bond acceptors, two hydrophobics and the rest are of the Excluded Volume type–spheres derived from the atomic coordinates of POP atoms. S3 Table presents the coordinates of these features.

We found that a minimum of 9 features is best as we tested a set of the 37 full inhibitors and 7,104 randomly picked molecules. More features did not affect the results. With that requirement, 4 out of 37 full inhibitors and 5 out of 7,104 random molecules were false positives. The condition of at least 9 features was used for subsequent tests of the models described below.

Four structure-based pharmacophore models were created by LigandScout 3.1 (Inte:ligand) [46] based on the described modifications of the 1E8N complex.

Three of these pharmacophore models are "automatic": 1) Each feature in the S1-S1' area is defined "Excluded Volume". It is featured as Excluded Volumes (FaEV); 2) Feature Removal (FR)–features in S1-S1' area, which are not "Excluded Volume" types, were removed; 3) Removal of P1-P1' (P1P1'R)–removing the Pro-Phe, which are P1-P1' positions, from the enzyme-substrate complex, and creating the pharmacophore model according to this modification. If a single conformation (out of fifty) was found to conform to one of the 3 methods, the ligand is considered as "positive" and assumed not to interact with the S1-S1' area.

The fourth method was Visual Inspection (VI), in which we visually examined each of the 50 conformations of each molecule for presence or absence in the S1-S1' area of POP (in this option we demand that at least 30 poses will not interact with the S1-S1’ area), a highly tedious and time consuming examination.

#### ISE classification modeling [26]

In the learning set we have actives and inactives (actives are diluted by at least 100-fold inactives). We refer to the ncP52 set as the actives, assuming that the fragments in that set could function as substrate selective inhibitors. The inactive set contains only random molecules. A large set (~200) of Physico-chemical descriptors for each molecule of the learning set were calculated by MOE 2011 [47]. To avoid bias, in cases of high correlation between two descriptors (r^2^ > 0.81), the descriptor which has a larger sum of r^2^ with all the other descriptors is eliminated.

The learning set was divided randomly into five subsets, each containing 1/5 of the actives and of the inactives. ISE was applied five times, to create a model based on 4 subsets as training and then applying that model to the remaining, one test set for scoring. At the end of this process, all learning set molecules have a score, having been once evaluated as a test set by a model created by the training set. The output of each fold is an ensemble of filters, while each filter is a combination of five ranges of physico-chemical descriptors. Filters of all five folds are combined, eliminating similar filters and ordering by their MCC (Eq 1) values.

The first step in each fold (a training set with 4/5 of the molecules) is to divide the range of each descriptor by 100, thus making 4950 internal ranges (n*(n-1)/2). All that fold's molecules are examined by each of the internal ranges of a descriptor to find which range supplies the best ability to distinguish actives form randoms. This is achieved by calculating the Matthews Correlation Coefficient (MCC) (Eq 1) [48], which is a major parameter for classifications, when two sets are very different in size. The pool of single most successful ranges for each of the descriptors is the basis for picking "filters" of 5 randomly picked descriptor ranges. The number of possible combinations for constructing 5-membered filters out of ~180 descriptors is about 1.5*10^9^. ISE has been demonstrated to deal with much greater complexity, even more than an initial huge number of 10^100^ alternatives [49].

$$MCC=\frac{TP*TN-FP*FN}{\sqrt{\left(TP+FP)*(TP+FN)*(TN+FP)*(TN+FN\right)}}$$ Eq 1

The algorithm picks randomly filters of 4–5 descriptor ranges and records how many molecules of the training set pass or fail to pass each filter, depending on their own descriptor values. A known active that passes all the filter's descriptor ranges is a True Positive (TP) but it is a False Positive (FP) if the molecule is an assumed inactive (randomly picked). A molecule that fails to pass a filter is a True Negative (TN) if it is an inactive, and is a False Negative (FN) if it is a known active.

The algorithm passes each molecule of the training set through each randomly picked filter and calculates the proportions of TP and FP as well as of TN and FN for the training set, for each filter that is randomly picked. These four numbers are the components of the MCC, which has values between -1 and 1, with larger positive numbers reflecting a better filter for distinguishing between actives and inactives (Eq 1).

Thus every generated filter has an associated MCC. Once a very large number of random filters has been generated, a virtual histogram presents the appearances of all MCC values. That large number assures that the optimal range of each descriptor appears similarly in the total sample, i.e., there is an “expectation value” for each descriptor which is similar for the whole range of MCC values. The number of appearances of a descriptor should be evenly spread over MCC values (from -1 to +1) only if there is no bias by being associated with good (high positive MCC) or bad (low negative MCC) values. Each MCC is associated with a specific filter and with its descriptor ranges, and it is now possible to evaluate the contribution of each descriptor range to the worst and to the best MCC values. We focus only on the worst 10% of MCC values and on the best 10% for assessing descriptor range involvement. Thus, ranges that appear much more than the expected average among the worst 10% MCC values, and appear much less than the average among the top 10% MCC values–are eliminated from the set of descriptor ranges. By that, the total number of ranges is reduced. A subsequent iteration of large random sampling of filters is conducted, leading to the next virtual histogram, assessment of the role of each remaining descriptor range and further eliminations. That process is repeated until the number of possible descriptor combinations is less than a million. From that point, an exhaustive computation of all possible descriptor combinations takes place–all remaining descriptors are combined into filters by a systematic process that allow to evaluate the performance of all remaining filters, score them and sort them.

Each filter gets an MCC value and the total filters may now be sorted. Once that ensemble of best solutions has been formed, the algorithm optimizes them by clustering. Filters are kept either on the basis of having an MCC not lower than 20% less than the top MCC, or, by keeping the top 1000 filters. Those constitute the model through which we virtually screen millions of molecules and pick the molecules which get top Indexes, which are the normalized final scores.

Screening of a huge molecular set (commercially available in the present case) is performed by adding the TP/FP value of a filter to each molecule that successfully passes that filter, or subtracting the value of TN/FN for each filter that the molecule does not pass. Each screened molecule accumulates the scores for all filters and the final Index is an average over all the filters. The equation for the Indexing of molecules is Eq 2:

$$MBI=\frac{\sum_{i=1}^{n}(\delta active\frac{P}{Pf}-\delta inactive\frac{N}{Nf})}{n}$$ Eq 2

While n is the total number of filters. δactive or δinactive will be 1 or 0 (depending on an active or inactive molecule predicted as positive or negative, respectively). P/P_f_ is the proportion of TP/FP in a particular filter and can be referred as an efficiency factor (while N/N_f_ is TN/FN and is an “inefficiency factor”).

#### Solubility calculations

We used five different programs to evaluate solubility. In addition to numbers given by Enamine and SciFinder [50] and the cLogS value of MOE, two additional tools were used for estimating the solubility of candidates: AdmetSAR [51] and VCCLAB [52] servers. Solubility evaluations were performed by computing averages of these 5 programs and using the standard deviations to decide whether an average is a reasonable representative and should be considered for further decisions.

#### Docking the candidates to the POP structure

Docking was performed in order to confirm the results of the kinetic studies. Each of the 20 candidates (50 conformations maximum each) was docked by Glide (Schrödinger) [34] with the SP (standard precision) algorithm. Docking experiments were performed for all 20 candidates, in order to examine the chances of each to be a competitive inhibitor for Ang-III and non-competitive inhibitor for TRH. For simulating that we used the positions of the known substrate in crystal structure 1E8N. This substrate extends between P5 to P2’ and in order to simulate the short peptide sequence of TRH, we “chopped off” the substrate positions P5 to P4 and examined whether the 20 inhibitor candidates could serve as non-competitive inhibitors by binding to S5-S4 positions. A grid of 5Å was used as “ligand reference” at P5-P4 ligand positions of 1E8N. Our assumption is that if a candidate binds at these positions–it is a competitive inhibitor for long substrates such as Ang-III. The ligand reference in this docking was P5-P4 positions of the crystallographic substrate.

### Experimental procedures

The in vitro experiments were performed with the human enzyme which has a sequence identity of 97.2% to the *sus scrofa* enzyme that we used for the in silico docking and pharmacophore studies (PDB ID: 1E8N). There are no significant differences around the active site as well as in more remote enzyme parts between the structures of these two enzymes (see S1 Table).

#### In vitro test for inhibition

Preparation of recombinant human POP (rhPOP) was as follows: Human POP cDNA was amplified by RT-PCR using total RNA extracted from human neuroblastoma NB-1 cells (IFO50295, Health Science Research Resources Bank, Osaka, Japan). The PCR products were cloned into pGEX-6P vectors (GE Healthcare) according to the manufacturer’s protocol. The encoded glutathione S-transferase (GST)-POP fusion proteins were expressed in E. coli strain BL-21 (GE Healthcare) and purified using a Glutathione Sepharose 4B column (GE Healthcare). After the GST moiety of the purified fusion protein was removed with PreScission Protease (GE Healthcare), rhPOP was finally purified using a Mono-Q 5/50 GL column (GE Healthcare).

The assay mixture containing 10 μL of inhibitor (at an indicated concentration) in DMSO, 10 μL of rhPOP (1–5 units) and 340 μL of 50 mM Tris-HCl buffer (pH7.2) were preincubated at 37°C for 5 min. One unit of POP is defined as the amount of enzyme needed to hydrolyze 1 nmol of succinyl-Gly-Pro-4-methylcoumarin-7-amide (Peptide institute, Inc. Osaka, Japan) per min under the assay condition. The control solution was prepared using the same buffer but without inhibitor. Afterwards, 40 μL of aqueous solution of substrate (1 mM of Ang-III or 10 mM TRH) was added to the reaction solution and control solution, individually. The reaction mixture was incubated at 37°C for 10 min. The reaction was terminated by adding 100 μL of trifluoroacetic acid (TFA).

The reaction mixtures were analysed using an HPLC equipped with C18 column (4.6 mm×150 mm, particle size 5 μm, Inertsil ODS-2, GL Science, Tokyo, Japan). The mixtures were separated using a linear gradient of acetonitrile (0–25% for TRH and its hydrolyzed products, 5–27.5% for Ang-III and its hydrolyzed products) in 0.05% TFA at a flow rate of 0.5 mL/min for 20 min. The resulting Ang III or TRH peptides were detected using a UV detector fixed at 214 nm.

#### POP-Inhibitory Activity Assay and Determination of IC_50_

First, the effect of 100 μM of inhibitor on the POP catalytic action toward Ang-III and TRH was investigated for 20 candidate inhibitors and a few randoms. Stock inhibitor solutions of 20 mM in DMSO were prepared and then diluted with DMSO to give a 4 mM. The degree of POP-residual activity (%) was calculated according to the following Eq (3):

$$POP\;residual\;activity(\%)=\frac{\Delta A\;sample}{\Delta A\;control}*100$$ Eq 3

where ΔA inhibitor sample and ΔA control were the peak areas of Ang-III fragment or TRH fragment in the samples with or without inhibitor, respectively.

Next, IC_50_ values were determined for 2 inhibitors (T5450157 and T6816369) that showed inhibitory activity. The IC_50_ is defined as the required concentration for 50% inhibition of POP activity. The IC_50_ was determined by nonlinear regression using GraphPad Prism 7 (GraphPad, San Diego, CA).

#### Determination of Inhibitory Kinetics

Kinetic parameters (V_max_ and K_m_) were estimated by fitting the Michaelis-Menten equations to the substrate concentrations and the initial reaction rates. Data analysis and curve fitting were performed with GraphPad Prism 7.

Then, the Dixon plot^35^ was used to determine the inhibition type of the inhibitors, which were competitive, noncompetitive or uncompetitive. Linear regression analysis of reciprocal saturable uptake (1/v) for different substrate concentrations (1.0 μM or 2.0 μM Ang III and 100 μM or 200 μM TRH) as a function of inhibitor concentration was performed.

## Supporting information

S1 Fig POP structure. Cartoon of the catalytic (orange) and the beta-propeller (yellow) domains is presented. Residues of the catalytic sites and the oxyanion hole are presented in brown sticks, with the catalytic triad in the upper part (Asp/His/Ser) and the Tyr of the oxyanion hole somewhat lower. The part of the substrate between P1 and P2' is in pink while the part from P1 to the N-terminal is green.
(TIF)

S2 Fig The elimination of fragments which are assumed to occupy the P1 position. A) The crystallographic inhibitors. P1-parallel Fragments position (P1PF) are shown in red. The subtitles are the relevant PDB codes. B) The cP52 set, resulting from the cleavage at P1PF. Similar fragments are connected by colored boxes. C) The non-crystallographic inhibitors. Fragments that are assumed to be P1PF are shown in red (one of them is in purple, as is the second option of CHEMBL363383). The subtitles are the originals from ChEMBL database. D) Respectively, the ncP52 set, following the cleavage of the fragments that are assumed P1PF. Similar fragments are connected by colored boxes.
(TIF)

S3 Fig A matrix of Tanimoto values between the cP52 set (the names of the source PDB are in the upper line) and the ncP52 set (the names of the ChEMBL sources are in the left column). As the Tanimoto value is higher, the number appears more black than gray. The maximum Tanimoto value is calculated for each molecule, last column (as the maximum value is higher, the number is marked in deeper blue). Four fragments are identical in these two sets.
(TIF)

S4 Fig Distinguishing between ncP52 and other sets by the ISE model. A) ROC curve of the ISE model (ncP52 vs. random molecules). B) The same curve replacing ncP52 fragments by the original inhibitors. The difference in AUC is huge (0.97 vs. 0.59) and indicates randomness of the results for the original inhibitors as True positives, while there is confidence in the results for the ncP52 fragments as True positives.
(TIF)

S5 Fig **Groups of molecules sent for in vitro tests from left to right:** A) Top pharmacophore candidates (2); B) Other pharmacophore candidates (6); C) Top ISE candidates (8); D) Other ISE candidates (4); E) Tanimoto only candidates with minimal MBI (3) F) Random molecules from Enamine (2).
(TIFF)

S6 Fig Reversed phase high pressure liquid chromatography (RP-HPLC) of Ang III and TRH peptides. Chromatograms (Absorption at 214 nm plotted against time) obtained by analyzing the reaction mixture of rhPOP and Ang III or TRH by RP-HPLC are shown. A) Chromatogram of the reaction mixture of rhPOP and Ang III. B) Chromatogram of the reaction mixture of rhPOP and TRH.
(TIF)

S7 Fig MALDI-TOF MS spectra of TRH (pGlu-His-Pro-NH2: 362.39 g/mol) at retention time 14.8 min (upper), and TRH-OH (pGlu-His-Pro-OH: 363.67 g/mol) at retention time 17.0 min (lower).
(TIF)

S8 Fig Measurements of IC_50_ values (for activity of Ang-III and TRH) in the presence of T6816369 and T5450157. (TIF)

S1 Table 15 complexes of POP-inhibitors. The organism source is indicated, as well as the RMSD with respect to 1E8N.
(PDF)

S2 Table Applicability domain calculation for choosing the set of inactives. Applicability domain is required in order to avoid the inclusion of learning set molecules that have very different properties than the "actives" (such as salt or huge molecules) and might therefore bias the modeling. Calculations are based upon the 174 active molecules from ChEMBL. For each of the descriptors representing Lipinski's rule of five the average and the standard deviations (σ) are calculated for the "actives". Random molecules must have the 4 properties within the range of the average plus/minus 2 standard deviations.
(PDF)

S3 Table Coordinates of the features in the pharmacophore model. (PDF)

S4 Table Number of molecules that passed the Pharmacophore test for each set, according to the different approaches. Lines are for the different sets of molecules, columns are for the different pharmacophore methods. In the case of the "Visual Inspection" strategy we specify whether there are more than 15 or more than 30. The last columns present the "consensus"—the number of molecules successful in each method and the number of molecules in the set.
(PDF)

S5 Table Detailed presentation for each molecule that passed one of the strategies and the overlap rate between the strategies. The molecules are sorted according to the percentage of the desirable conformations out of all the conformations that are supplied by the program in the "Visual Inspection" strategy. The last column shows if the molecule succeeded or not according to all the approaches.
(PDF)

S6 Table Number of molecules from different sets that are above the ISE MBI cutoff. Columns, left to right: Cutoffs of MBI; ncP52 fragments; ChEMBL inhibitors; random molecules from the learning set (ncRandom); unique cP52 fragments (cP52); X-ray inhibitors; Random molecules from the external test set (cRandom); and initial candidate SSIs.
(PDF)

S7 Table MBI values of the unique cP52 fragments and of their original inhibitors in the ISE model. The maximum Tanimoto values between these fragments and the ncP52 set are given in the right column.
(PDF)

S8 Table Inhibition results (percent) by all 25 molecules. The first column presents the method by which molecules were selected, second column presents the Enamine molecular identification, third and fourth columns present the percent inhibition of POP activity in presence of the two substrates Ang-III and TRH.
(PDF)
