# Supplementary figures and images for: Computational design of substrate selective inhibition
Source: PLoS Comput Biol. 2020 Mar 20;16(3):e1007713. doi: 10.1371/journal.pcbi.1007713 (PMC7112232; doi:10.1371/journal.pcbi.1007713)

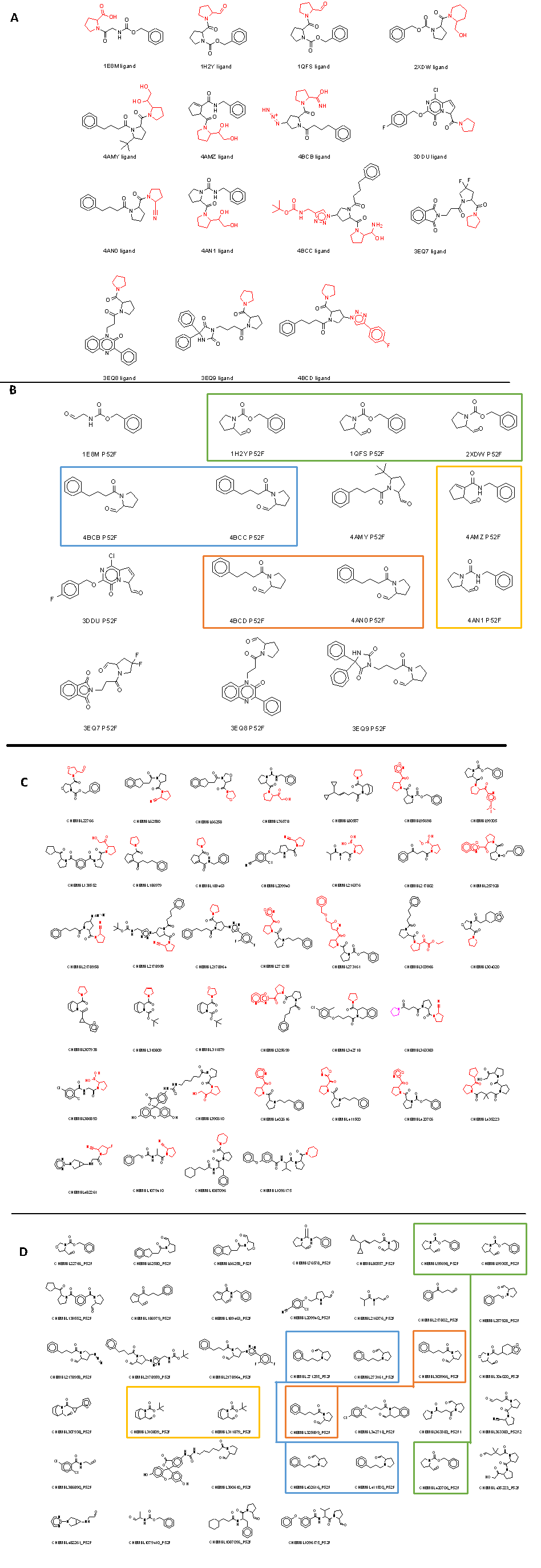

Supplement: S2 Fig — A) The crystallographic inhibitors. P1-parallel Fragments position (P1PF) are shown in red. The subtitles are the relevant PDB codes. B) The cP52 set, resulting from the cleavage at P1PF. Similar fragments are connected by colored boxes. C) The non-crystallographic inhibitors. Fragments that are assumed to be P1PF are shown in red (one of them is in purple, as is the second option of CHEMBL363383). The subtitles are the originals from ChEMBL database. D) Respectively, the ncP52 set, following the cleavage of the fragments that are assumed P1PF. Similar fragments are connected by colored boxes. (TIF) [file pcbi.1007713.s002.tif]

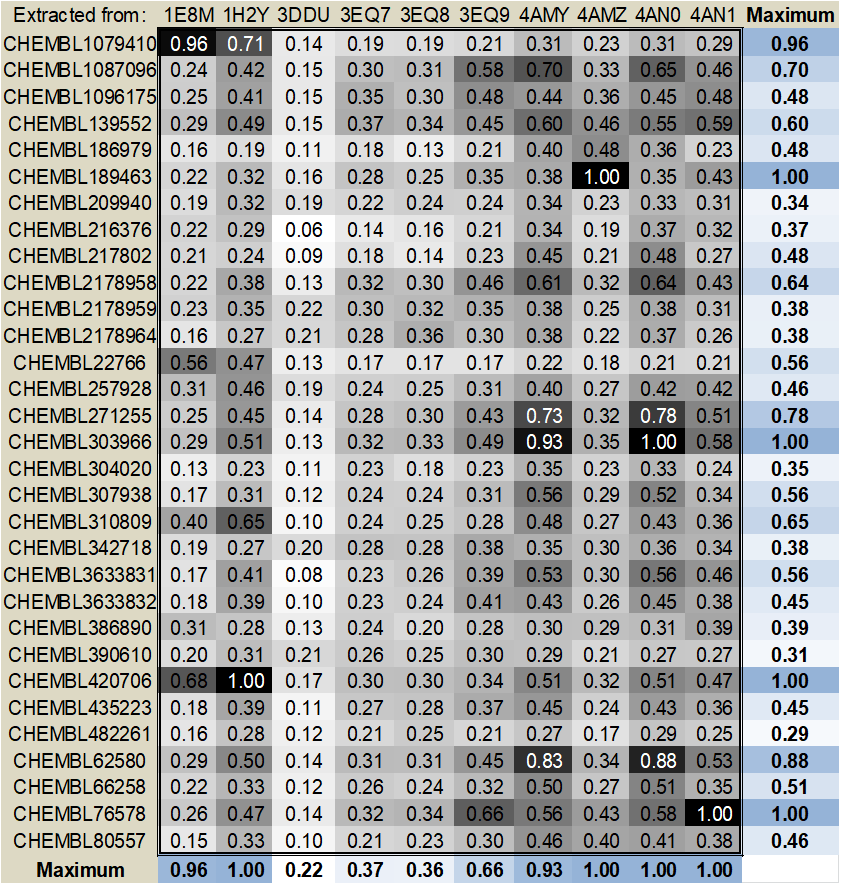

Supplement: S3 Fig — A matrix of Tanimoto values between the cP52 set (the names of the source PDB are in the upper line) and the ncP52 set (the names of the ChEMBL sources are in the left column). As the Tanimoto value is higher, the number appears more black than gray. The maximum Tanimoto value is calculated for each molecule, last column (as the maximum value is higher, the number is marked in deeper blue). Four fragments are identical in these two sets. (TIF) [file pcbi.1007713.s003.tif]

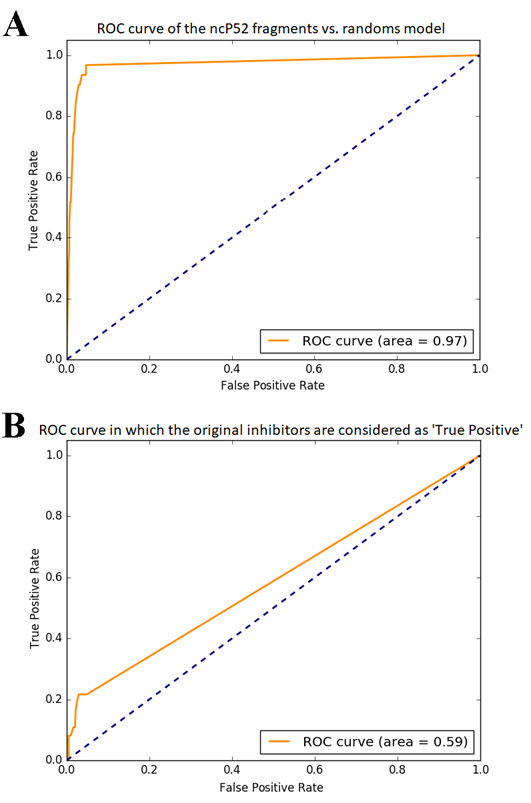

Supplement: S4 Fig — A) ROC curve of the ISE model (ncP52 vs. random molecules). B) The same curve replacing ncP52 fragments by the original inhibitors. The difference in AUC is huge (0.97 vs. 0.59) and indicates randomness of the results for the original inhibitors as True positives, while there is confidence in the results for the ncP52 fragments as True positives. (TIF) [file pcbi.1007713.s004.tif]

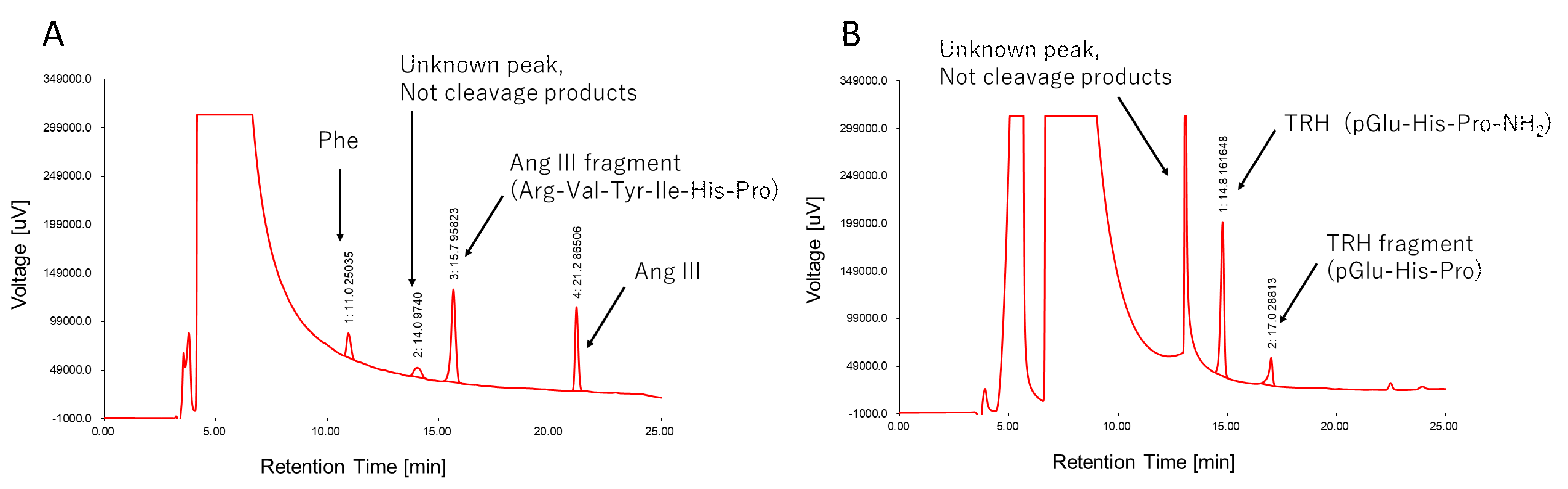

Supplement: S6 Fig — Chromatograms (Absorption at 214 nm plotted against time) obtained by analyzing the reaction mixture of rhPOP and Ang III or TRH by RP-HPLC are shown. A) Chromatogram of the reaction mixture of rhPOP and Ang III. B) Chromatogram of the reaction mixture of rhPOP and TRH. (TIF) [file pcbi.1007713.s006.tif]

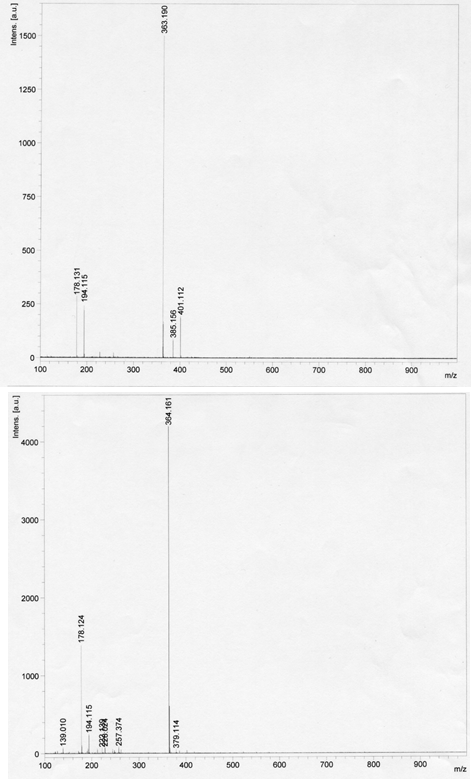

Supplement: S7 Fig — MALDI-TOF MS spectra of TRH (pGlu-His-Pro-NH2: 362.39 g/mol) at retention time 14.8 min (upper), and TRH-OH (pGlu-His-Pro-OH: 363.67 g/mol) at retention time 17.0 min (lower). (TIF) [file pcbi.1007713.s007.tif]

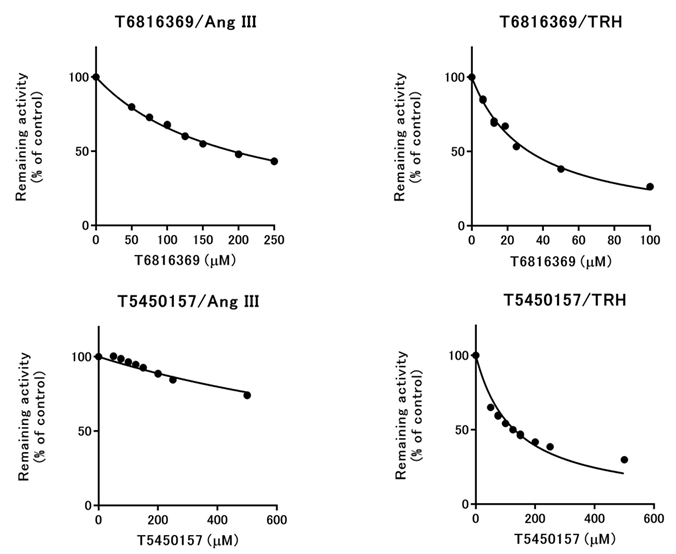

Supplement: S8 Fig — (TIF) [file pcbi.1007713.s008.tif]
