# Supplementary material for: Computational design of substrate selective inhibition
Source: PLoS Comput Biol. 2020 Mar 20;16(3):e1007713. doi: 10.1371/journal.pcbi.1007713 (PMC7112232; doi:10.1371/journal.pcbi.1007713)
Supplement: S1 Table — The organism source is indicated, as well as the RMSD with respect to 1E8N. (PDF) [file pcbi.1007713.s009.pdf]

Complex	Source	RMSD to 1E8N
1QFS	Sus scrofa	0.31
1E8M	Sus scrofa	0.28
1H2Y	Sus scrofa	0.21
2XDW	Sus scrofa	0.28
3DDU	Homo sapiens	0.31
3EQ7	Sus scrofa	0.37
3EQ8	Sus scrofa	0.34
3EQ9	Sus scrofa	0.35
4AMY	Sus scrofa	0.28
4AMZ	Sus scrofa	0.27
4AN0	Sus scrofa	0.27
4AN1	Sus scrofa	0.31
4BCB	Sus scrofa	0.26
4BCC	Sus scrofa	0.29
4BCD	Sus scrofa	0.30
