# Supplementary material for: Computational design of substrate selective inhibition
Source: PLoS Comput Biol. 2020 Mar 20;16(3):e1007713. doi: 10.1371/journal.pcbi.1007713 (PMC7112232; doi:10.1371/journal.pcbi.1007713)
Supplement: S2 Table — Applicability domain is required in order to avoid the inclusion of learning set molecules that have very different properties than the "actives" (such as salt or huge molecules) and might therefore bias the modeling. Calculations are based upon the 174 active molecules from ChEMBL. For each of the descriptors representing Lipinski's rule of five the average and the standard deviations (σ) are calculated for the "actives". Random molecules must have the 4 properties within the range of the average plus/minus 2 standard deviations. (PDF) [file pcbi.1007713.s010.pdf]

Descriptor	lip_acc	lip_don	logP(o/w)	Weight
AVERAGE	6.21	0.42	2.36	405.47
STDEV (σ)	1.8	0.83	1.16	73.18
Range	2.6-9.82	0-2.09*	0.05-4.67	259.12-551.83

* In the case of lip_don the range should be -1.25 to 0.9. Yet, negative values for Hydrogen bond donors are impossible
