# Supplementary material for: Computational design of substrate selective inhibition
Source: PLoS Comput Biol. 2020 Mar 20;16(3):e1007713. doi: 10.1371/journal.pcbi.1007713 (PMC7112232; doi:10.1371/journal.pcbi.1007713)
Supplement: S3 Table — (PDF) [file pcbi.1007713.s011.pdf]

Type		Coordinates		
		X	Y	Z
Hydrophobic		37.80	37.60	87.28
Hydrophobic		45.42	35.40	87.76
Hydrogen Bond Acceptor	origin (→)	42.09	36.17	81.67
	target (O)	42.88	37.55	84.13
Hydrogen Bond Acceptor	origin (→)	40.47	41.51	85.40
	target (O)	39.55	39.14	84.14
Hydrogen Bond Acceptor	origin (→)	34.69	36.46	83.94
	target (O)	35.76	38.13	82.13
Hydrogen Bond Acceptor	origin (→)	34.16	37.87	79.54
	target (O)	35.76	38.13	82.13
Hydrogen Bond Donor	origin (→)	35.32	41.79	83.42
	target (O)	37.43	42.64	81.71
Excluded Volume		42.09	36.17	81.67
Excluded Volume		40.47	41.51	85.40
Excluded Volume		42.20	33.43	85.34
Excluded Volume		34.69	36.46	83.94
Excluded Volume		45.60	33.37	83.26
Excluded Volume		37.43	42.64	81.71
Excluded Volume		48.08	37.03	83.60
Excluded Volume		34.58	35.56	89.46
Excluded Volume		34.16	37.87	79.54
Excluded Volume		45.58	29.92	85.34
Excluded Volume		34.48	40.25	78.96
Excluded Volume		49.75	35.62	87.66
Excluded Volume		48.33	41.44	89.62
Excluded Volume		50.13	41.97	92.91
