# Supplementary material for: Computational design of substrate selective inhibition
Source: PLoS Comput Biol. 2020 Mar 20;16(3):e1007713. doi: 10.1371/journal.pcbi.1007713 (PMC7112232; doi:10.1371/journal.pcbi.1007713)
Supplement: S4 Table — Lines are for the different sets of molecules, columns are for the different pharmacophore methods. In the case of the "Visual Inspection" strategy we specify whether there are more than 15 or more than 30. The last columns present the "consensus"—the number of molecules successful in each method and the number of molecules in the set. (PDF) [file pcbi.1007713.s012.pdf]

Strategy	Visual Inspection Any	Visual Inspection >15	Visual Inspection > 30	P1-P1' Removal	Features as EV *	Features Removal	Consensus	Out of molecules
Initial candidate SSIs	47	38	30	10	22	22	11	11,704
original inhibitors	4	2	2	2	3	3	1	37
ncP52 fragments	11	8	7	7	8	8	6	31
random molecules	5	4	2	2	1	2	0	7,104
