# Supplementary material for: Computational design of substrate selective inhibition
Source: PLoS Comput Biol. 2020 Mar 20;16(3):e1007713. doi: 10.1371/journal.pcbi.1007713 (PMC7112232; doi:10.1371/journal.pcbi.1007713)
Supplement: S5 Table — The molecules are sorted according to the percentage of the desirable conformations out of all the conformations that are supplied by the program in the "Visual Inspection" strategy. The last column shows if the molecule succeeded or not according to all the approaches. (PDF) [file pcbi.1007713.s013.pdf]

Set	Strategy	Visual Inspection		Blind methods			Consensus
	molecule/fragment	"Successful poses"	%	P1-P1' Removal	Features as EV	Features Removal	
ncP52	CHEMBL216376_P52F	46	92.00	Yes	Yes	Yes	Yes
	CHEMBL217802_P52F	45	90.00	Yes	Yes	Yes	Yes
	CHEMBL62580_P52F	44	88.00	Yes	Yes	Yes	Yes
	CHEMBL186979_P52F	38	76.00	Yes	Yes	Yes	Yes
	CHEMBL420706_P52F	32	64.00	Yes	Yes	Yes	Yes
	CHEMBL325699_P52F	32	64.00	Yes	Yes	Yes	Yes
	CHEMBL363383_P52F1	31	62.00				No
	CHEMBL307938_P52F	17	42.50		Yes	Yes	No
	CHEMBL310809_P52F	5	33.33	Yes	Yes	Yes	No
	CHEMBL363383_P52F2	13	32.50				No
	CHEMBL257928_P52F	7	14.00				No
original Inhibitors	CHEMBL186979	50	100.00	Yes	Yes	Yes	Yes
	CHEMBL3079383	50	100.00	Yes			No
	CHEMBL363383	14	28.00		Yes	Yes	No
	CHEMBL310809	0	0.00		Yes	Yes	No
Random molecules	T6990286	32	88.89				No
	T5219748	32	64.00				No
	T7019965	22	44.00	Yes		Yes	No

	T6483240	19	38.00	Yes	Yes	Yes	No
	T6865906	6	23.08				No
	T6187808	50	100	Yes	Yes	Yes	Yes
	T6187809	50	100	Yes	Yes	Yes	Yes
	T6436039	50	100	Yes	Yes	Yes	Yes
	T6571969	50	100	Yes	Yes	Yes	Yes
	T6696582	50	100	Yes	Yes	Yes	Yes
	T6708537	50	100	Yes	Yes	Yes	Yes
	T6716851	50	100	Yes	Yes	Yes	Yes
	T6765795	50	100	Yes	Yes	Yes	Yes
	T6816369	50	100	Yes	Yes	Yes	Yes
	T6939594	50	100		Yes	Yes	no
Initial candidate SSIs	T6948193	50	100		Yes	Yes	no
	T6949653	50	100		Yes	Yes	no
	T6964073	50	100		Yes	Yes	no
	T6836819	43	100		Yes	Yes	no
	T5584022	35	100		Yes	Yes	no
	T7003616	18	100		Yes	Yes	no
	T6782356	11	100		Yes	Yes	no
	T6787071	9	100		Yes	Yes	no
	T6890951	49	98				no
	T7026561	49	98				no

T6919782	28	97				no
T6803674	48	96	Yes	Yes	Yes	Yes
T6988334	47	94				no
T6876613	38	93	Yes	Yes	Yes	Yes
T6360132	46	92				no
T6877907	44	88				no
T6988147	44	88				no
T6782323	43	86				no
T5221497	42	84				no
T7100354	26	84	Yes			no
T6552942	41	82				No
T6518405	38	76				No
T6648206	36	72				No
T6131200	34	68				No
T6883177	33	66				No
T6046367	29	58				No
T6148935	29	58				No
T5646202	27	54				No
T5848618	25	50				No
T6117285	6	50				No
T6120909	22	44				No
T5887431	4	17				No
T6436022	7	14				No

T6187807	3	6	Yes	Yes	Yes	No
T6192791	2	4				No
T6436019	0	0	Yes			No
T5656239	0	0				No
T7088399	-	-	Yes			No
