# Supplementary material for: Computational design of substrate selective inhibition
Source: PLoS Comput Biol. 2020 Mar 20;16(3):e1007713. doi: 10.1371/journal.pcbi.1007713 (PMC7112232; doi:10.1371/journal.pcbi.1007713)
Supplement: S6 Table — Columns, left to right: Cutoffs of MBI; ncP52 fragments; ChEMBL inhibitors; random molecules from the learning set (ncRandom); unique cP52 fragments (cP52); X-ray inhibitors; Random molecules from the external test set (cRandom); and initial candidate SSIs. (PDF) [file pcbi.1007713.s014.pdf]

Cutoff	ncP52	original inhibitors from ChEMBL	ncRandoms	cP52	original inhibitors of X-ray	cRandoms	Initial candidate SSIs
> -1	31	37	7104	6	6	5450	11,714
> -0.9	30	8	345	6	0	313	947
> -0.8	30	8	334	6	0	313	946
> -0.7	29	8	328	6	0	313	943
> -0.6	29	8	297	6	0	312	915
> -0.5	29	8	283	6	0	299	861
> -0.4	29	8	258	6	0	280	783
> -0.3	28	8	225	6	0	260	619
> -0.2	28	8	206	6	0	239	474
> -0.1	27	7	174	6	0	200	352
> 0	26	6	153	6	0	162	228
> 0.1	25	4	143	6	0	131	136
> 0.2	23	4	124	5	0	103	95
> 0.3	23	4	113	5	0	80	84
> 0.4	16	3	70	5	0	69	65
> 0.5	16	3	60	4	0	51	52
> 0.6	14	3	45	3	0	37	38
> 0.7	11	3	43	3	0	21	28
> 0.8	10	3	33	3	0	14	17
>0.85	10	1	33	3	0	14	13

> 0.9	10	0	33	2	0	7	11
