# Supplementary material for: Computational design of substrate selective inhibition
Source: PLoS Comput Biol. 2020 Mar 20;16(3):e1007713. doi: 10.1371/journal.pcbi.1007713 (PMC7112232; doi:10.1371/journal.pcbi.1007713)
Supplement: S7 Table — The maximum Tanimoto values between these fragments and the ncP52 set are given in the right column. (PDF) [file pcbi.1007713.s015.pdf]

PDB code	Original inhibitor (MBI)	cP52 fragment (MBI)	Maximum tanimoto for the ncP52 set
1E8M	-0.971	0.914	0.96
3DDU	-0.972	0.172	0.22
3EQ7	-0.972	0.512	0.37
3EQ8	-0.971	0.895	0.36
3EQ9	-0.971	0.933	0.66
4AMY	-0.971	0.475	0.93
