# Supplementary material for: Computational design of substrate selective inhibition
Source: PLoS Comput Biol. 2020 Mar 20;16(3):e1007713. doi: 10.1371/journal.pcbi.1007713 (PMC7112232; doi:10.1371/journal.pcbi.1007713)
Supplement: S8 Table — The first column presents the method by which molecules were selected, second column presents the Enamine molecular identification, third and fourth columns present the percent inhibition of POP activity in presence of the two substrates Ang-III and TRH. (PDF) [file pcbi.1007713.s016.pdf]

Selected by	Candidate	ANG-III	TRH
Pharmacophore	T6187808	5.3	7.2
Pharmacophore	T6816369	41.4	75
Pharmacophore	T6939594	3.7	5.1
Pharmacophore	T7003616	5.1	1
Pharmacophore	T6890951	4.9	4
Pharmacophore	T7100354	0	2.3
Pharmacophore	T6436019	4.2	7.9
Pharmacophore	T7088399	9.3	12.6
ISE	T5606955	4.3	6.1
ISE	T5450157	10.5	43
ISE	T5638984	3.2	1.3
ISE	T5752573	3.7	4.3
ISE	T6614961	1.9	0.6
ISE	T6669772	2.8	6.9
ISE	T5727123	11	18.5
ISE	T6553333	1.7	1.2
ISE	T6827388	5.9	16
ISE	T5434643	0.4	5.2
ISE	T5689739	4.2	6.5
ISE	T6416783	4.4	1.9
Non-candidate	T5936039	0	0

Non-candidate	T6188448	0	5.6
Non-candidate	T6293431	0	0.4
Non-candidate	T5545481	0	0
Non-candidate	T5420863	0	0.8
